# Microbiota is essential for social development in the mouse

**DOI:** 10.1038/mp.2013.65

**Published:** 2013-05-21

**Authors:** L Desbonnet, G Clarke, F Shanahan, T G Dinan, J F Cryan

**Affiliations:** 1 Alimentary Pharmabiotic Centre, University College Cork, Cork, Ireland; 2 Department of Psychiatry, University College Cork, Cork, Ireland; 3 Department of Medicine, University College Cork, Cork, Ireland; 4 Department of Anatomy and Neuroscience, University College Cork, Cork, Ireland

**Keywords:** Social neuroscience, Microbiota

The microbiota–gut–brain axis is an emerging concept in modern medicine informed by the ability of gut microbiota to alter brain and behaviour.^1^ Although some clinical studies have revealed altered gut microbiota composition in patients with neurodevelopmental disorders such as autism,^2, 3^ the specific contributions of microbiota in early life to the development and programming of the various facets of social behaviour has not been investigated.

Germ-free (GF) mice have been critical in assessing the role of microbiota in all aspects of physiology. Indeed, recent studies in GF mice report increases in neuroendocrine responses to stress,^4, 5^ altered neurotrophin levels in the hippocampus and amygdala,^5, 6, 7^ reduced anxiety^5, 6, 7^ and non-spatial memory,^8^ and altered monoamine neurotransmitter levels in the brain.^5, 6^ Interestingly, many of the deficits are specific to males^4^ in which there are higher incidence rates of neurodevelopmental disorders relative to females. Here, we examined the effects of GF rearing conditions through early life and adolescence on social behaviour in adulthood.

Mice, like humans, are a social species and have a natural propensity to seek out the security and pleasure afforded by stable social scenarios. Social motivation and preference for social novelty in mice can be assessed in the three-chambered sociability test.^9^ Our initial findings in this test revealed significant social impairments in GF mice, particularly in males, as indicated by a lack of the normal preference for time spent in a chamber containing a mouse versus the alternative empty chamber (GF × chamber interaction: F(1,57)=5.35, *P*<0.05; Supplementary Figures 1a–c). This was accompanied by reduced preference for novel social situations, where GF mice did not demonstrate the normal increase in time spent investigating a novel over a familiar mouse, which resembles social cognition deficits observed in patients with neurodevelopmental disorders (GF × chamber interaction: F(1,57)=5.86, *P*<0.01; Supplementary Figures 1d–f).

To substantiate these results and to assess the capacity for post-weaning bacterial colonisation of the GF gut (GFC) to reverse the observed social deficits, we repeated the test in a different male cohort. As expected, GF mice exhibited robust deficits including social avoidance (GF × chamber interaction: F(1,20)=12.41, *P*<0.001; Figures 1a–c), and diminished preference for social novelty relative to conventionally colonised (CC) mice (GF × chamber interaction: F(1,20)=4.45, *P*<0.05; Figures 1d–f). These effects were not influenced by changes in general locomotor activity, as any decrease in chamber entries was specific to the social chamber (Supplementary Figure 2). Intriguingly, whereas GFC reversed the observed social avoidance, it had no effect on social cognition impairments. This indicates that although the effects of GF rearing on the latter behaviour are permanently established in the pre-weaning period, the development of social avoidance in GF mice is more amenable to microbial-based interventions in later life.

**Figure 1:**
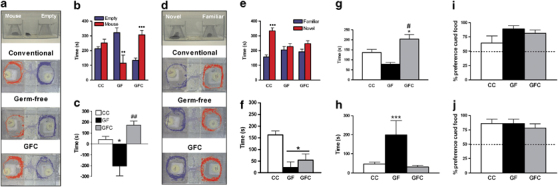
Effects of germ-free (GF) rearing and germ-free bacterial colonisation (GFC) on social behaviours in male mice. In the three-chambered sociability test, GF mice failed to show the normal preference for the social chamber displayed by conventionally colonised (CC) and GFC groups during trial 2, as seen in the automated tracking images (**a**), the time spent in each chamber (**b**) and the difference between time spent in mouse and empty chambers (**c**). This social avoidance was reversed in GFC mice (**b** and **c**). GF and GFC mice also failed to show normal preference for social novelty displayed by CC mice during trial 3, as seen in the automated tracking images (**d**), the time spent in each chamber (**e**) and the difference between time spent in the chambers containing a novel and familiar mouse (**f**). In the social transmission of food preference test, GF rearing conditions altered social investigation time (**g**) and grooming time (**h**) during social interaction with demonstrator mice. There was no effect on the preference for cued food immediately after social interaction (**i**) and 24 h later (**j**). ^••^*P*<0.01, ^•••^*P*<0.001 versus opposite chamber; repeated-measures analysis of variance followed by *post-ho*c Newman–Keuls test (*n*=7−13); *P<0.05, ***P<0.001 versus CC; ^#^*P*<0.01, ^##^*P*<0.001 versus GF; one-way analysis of variance followed by *post-ho*c Newman–Keuls test (*n*=5–13). PowerPoint slide

In addition to symptoms of reduced social motivation, children with autism exhibit poor social and communication skills and repetitive behaviours. To establish whether the degree of information transfer during social interaction is disrupted in GF mice, performance in the social transmission of food preference test was assessed. GF mice spent a decreased proportion of time engaged in social investigation (F(2,20)=7.51, *P*<0.005; Figure 1g) and substantially greater proportion of time engaged in repetitive self-grooming behaviour (F(2,20)=11.91, *P*<0.001; Figure 1h) during social interaction. These behaviours were normalised following GF bacterial colonisation, confirming the involvement of microbiota in modulation of these behaviours. However, despite the reduction in social investigation times, the quality of information transfer during the interaction was not affected in GF mice, as they displayed normal preference for the novel food (food to which cage-mate was exposed prior to social interaction) in the subsequent food choice test conducted immediately after the social interaction and 24 h later (Figures 1i and j), indicating that the ability to process information *per se* during social interaction is not affected in GF mice.

This study shows for, what is to our knowledge, the first time that microbiota are crucial for the programming and presentation of distinct normal social behaviours, including social motivation and preference for social novelty, while also being important regulators of repetitive behaviours. Given that these facets of behaviour are impaired in neurodevelopmental disorders such as schizophrenia and autism^10^ and with a similar male preponderance, these data may have implications for our understanding of the genesis of neurodevelopmental disorders of altered sociability. A better understanding of the mechanisms underlying these social deficits, which may include modulation of immune cell cytokines release, changes in vagal nerve activity and neuroendocrine function, could potentially lead to the emergence of novel and more effective therapies to combat symptoms in the social domain.

## Supplementary information

Supplementary Information (DOC 42 kb)

Supplementary Figure 1 (JPG 5781 kb)

Supplementary Figure 2 (JPG 1167 kb)

## PowerPoint slides

PowerPoint slide for Fig. 1
