# Supplementary material for: Microbiota is essential for social development in the mouse
Source: Mol Psychiatry. 2013 May 21;19(2):146–8. doi: 10.1038/mp.2013.65 (PMC3903109; doi:10.1038/mp.2013.65)
Supplement: Supplementary file 1 — Supplementary Information (DOC 42 kb) [file 41380_2014_BFmp201365_MOESM345_ESM.doc]

**Microbiota is Essential for Social Development in the Mouse: Relevance to Autism**

L. Desbonnet, G. Clarke, F. Shanahan, T.G. Dinan, J.F. Cryan

**Methods**

**Animals**

First-generation offspring from germ-free (GF) and conventionally-colonised (CC) Swiss Webster breeding pairs obtained from Taconic (Germantown, New York, USA) and were used in all experiments. GF Swiss Webster mice were housed 2-4/cage in flexible film gnotobiotic isolators under a strict 12-h light/dark cycle. Germ free colonised (GFC) mice were removed from the GF unit after weaning (postnatal day 21) and housed next to CC mice in the standard animal facility to allow microbes present in the environment to colonize. CC mice were similarly housed 4–5/cage in the standard animal facility under the same controlled conditions (temperature 20–21 °C, 55–60% humidity) on the same 12 h light/dark cycle. GF, GFC and CC mice received the same autoclaved pelleted diet (sodium dodecyl sulphate diets, product code 801010). All mice were tested in adulthood (postnatal day 55-60). Male and female NIH Swiss outbred mice (Harlan, UK) were used for social interaction and sociability/social novelty preference tests. Experiments were conducted in accordance with the European Directive 86/609/EEC and the Recommendation 2007/526/65/EC, and were approved by the Animal Experimentation Ethics Committee of University College Cork.

**Experimental design**

*Experiment 1*

GF mouse cages were removed from the sterile isolators and placed into a separate room with the CC mouse cages for at least 30 minutes prior to social behavioural testing. Male CC (n=9), female CC (n=7), male GF (n=6) and female GF (n=7) were tested in the sociability and social novelty preference test. 24hrs later social behaviours were assessed in the dyadic social interaction test.

*Experiment 2*

GF mouse cages were removed from the sterile isolators and placed into a separate room with the CC mouse cages for at least 30 minutes prior to social behavioural testing. Male CC (n=8), female CC (n=6), male GF (n=5), female GF (n=10), male GF-recolonised (GFR; n=13) and female GFR (n=13) were tested in the sociability and social novelty preference test. 24hrs later performance in the social transmission of food preference test was assessed. Mice were sacrificed and tissue processed (as described above) the day after the final behavioural test (48-52 hours after removal from the GF unit).

**Behavioural assessments**

*Sociability and social novelty preference test*

Preference for social contexts and social novelty was assessed as described previously (O’Tuathaigh et al., 2007; Yang et al., 2011). Briefly, mice were placed in a rectangular apparatus (36x20x20 cm) divided into 3 chambers (left and right chambers 13.5×20×20 cm; center chamber 9×20×20 cm) by transparent partitions with small circular openings allowing easy access to all compartments. The test was composed of 3 sequential 10 minute trials; trial 1: habituation (the mouse was allowed to explore the 3 chambers), trial2: sociability (an unfamiliar mouse was placed into a mesh wire cage in either the left or right chambers and exploration by the test mouse of the 3 chambers was recorded for a further 10 minutes), trial 3: social novelty preference (a novel mouse was placed into a mesh wire cage in the chamber opposite the (now familiar) mouse from the previous stage. Exploration of the 3 chambers by the test mouse was again recorded for 10 minutes. Stimulus mice used in this test were of the NIH Swiss strain, ordered from Harlan UK and housed in the facility for 4 weeks prior to testing. All mice used in the test were age- and sex-matched and each chamber was cleaned and lined with fresh bedding between trials. For each of the 3 stages, behaviours were recorded by a video camera mounted above the apparatus, and the Ethovision automated tracking system (Tracksys, UK) was used to analyze the time spent in, and frequency of entry into, each of the chambers (Feyder et al., 2010).

*Social transmission of food preference test*

Mice were housed in groups (2-4/cage). 18hrs prior to testing, mice were deprived of food, whereas water was available *ad libitum*. Mice were habituated to the square plastic containers (5 × 5 × 3cm) used to serve flavoured food choices in the test by placing one into each cage overnight. Food choices consisted of either 1% ground cinnamon or 2% powdered cocoa (Drinking Chocolate, Cadbury Ltd.) made with ground mouse chow. During testing, food flavours were alternated evenly within groups. These two flavoured diets possess equivalent metabolic and physical features (Clipperton et al., 2008) and proved equipalatable to mice housed in the same facility (data not shown).

*Demonstrator food choice:* A demonstrator mouse was randomly selected from each cage and the fur was marked using blue marker to enable identification during subsequent social interactions. The remaining mice in each cage acted as observers. Each demonstrator mouse was placed individually into clean polyethylene cages (20 × 20 × 30 cm; identical to the home cage) containing fresh bedding and a container with either 1% cinnamon mix or 2% cocoa mix and left for 1hr. Demonstrator food containers were weighed before and after the 1hr sampling sessions. A minimum of 0.2 g of consumed food was required for inclusion in the test.

*Social interaction:* Demonstrator mice were placed back into their respective home cages for a 20 minute interaction with cage-mates. Social interactions took place in the absence of food and water and were recorded from above using a video camera for later analysis.

*Observer food choice:* Observer mice were placed individually into clean polyethylene cages (identical to the home cage) containing fresh bedding and two containers with a choice between 1% cinnamon diet and 2% cocoa diet placed at opposite ends of the cage. Mice were left for 20 minutes and choice sessions were recorded from above for later analysis. Positions of flavoured diet containers were counterbalanced throughout the experiment to control for any position effect. Containers were weighed immediately before and after each choice session. Observer mice were then placed back into their respective home cages and the choice session was repeated 24hrs later.

**Statistics**

Multivariate analysis of variance was employed to assess group differences across data from the dyadic social interaction and STFP social interaction tests using SPSS 20.0. Repeated measures ANOVA were performed to analyse data from sociability/social novelty preference test [between subjects factors: group, sex (experiment 1 only); within subjects factor: chamber time), and retention of food preference in the STFP test (main factors: group, and time). Statistically significant effects in each ANOVA were followed with *post hoc* comparisons, using the Newman-Keuls test. A *p*-value of less than 0.05 was considered significant.

**References**

Clipperton A.E., Spinato J.M., Chernets C., Pfaff D.W., Choleris E. *Neuropsychopharmacol.* **33**, 2362-2375.

Desbonnet L, O'Tuathaigh C, Clarke G, O'Leary C, Petit E, Clarke N, Tighe O, Lai D, Harvey R, Cryan JF, Dinan TG, Waddington JL. *Brain Behav. Immun.* **26**, 660-671 (2012).

Feyder M, Karlsson RM, Mathur P, Lyman M, Bock R, Momenan R, Munasinghe J, Scattoni ML, Ihne J, Camp M, Graybeal C, Strathdee D, Begg A, Alvarez VA, Kirsch P, Rietschel M, Cichon S, Walter H, Meyer-Lindenberg A, Grant SG, Holmes A. Association of mouse Dlg4 (PSD-95) gene deletion and human DLG4 gene variation with phenotypes relevant to autism spectrum disorders and Williams' syndrome. *Am J Psychiatry* 2010;**167**, 1508-1517.

O'Tuathaigh CM, Babovic D, O'Sullivan GJ, Clifford JJ, Tighe O, Croke DT, Harvey R, Waddington JL. Phenotypic characterization of spatial cognition and social behavior in mice with 'knockout' of the schizophrenia risk gene neuregulin 1. *Neuroscience* 2007;**147**, 18-27.

Yang M, Silverman JL, Crawley JN. Automated three-chambered social approach task for mice. *Curr Protoc Neurosci* 2011; Chapter 8:Unit 8.26.

**Supplementary Figure 1:** Effects of germ-free (GF) rearing on social behaviours in the male and female mouse. In the 3-chambered sociability and social novelty preference test GF male mice failed to show the normal preference for the mouse chamber displayed by conventionally-colonised (CC) mice during trial 2, as seen in the automated tracking images **(a)**, the time spent in each chamber (b) and the difference between time spent in mouse and empty chambers (c) during trial 2. GF male mice also failed to show the normal preference for social novelty during trial 3 as seen in the automated tracking images **(d)**, the time spent in each chamber (e) and the difference between time spent in the chambers containing a novel and familiar mouse (f) during trial 3. * *p*<0.05 *versus* CC mice of the same sex; ••*p*<0.01, •••*p*<0.001 *versus* opposite chamber; repeated measures analysis of variance followed by *post-ho*c Newman-Keuls test (n = 7-9).

**Supplementary Figure 2:** Effects of germ-free (GF) rearing on chamber entries in the 3-chambered sociability and social novelty preference test. In experiment 1, GF male mice failed to show the normal preference for the mouse chamber displayed by conventionally-colonised (CC) mice during trial 2 (a). No difference in chamber entries was observed in GF mice of either sex in trial 3 (b). In experiment 2, male GF mice made fewer entries into the mouse chamber than the empty chamber, whereas, bacterial colonised GF mice (GFC) more frequently entered the mouse chamber than the empty chamber during trial 2 (c). As in experiment 1, there was no effect of GF rearing condition on chamber entries during trial 3 in experiment 2 (d). **p*<0.05 *versus* opposite chamber; repeated measures analysis of variance followed by *post-ho*c Newman-Keuls test (n = 7-13).
