# Supplementary figures and images for: Microbiota is essential for social development in the mouse
Source: Mol Psychiatry. 2013 May 21;19(2):146–8. doi: 10.1038/mp.2013.65 (PMC3903109; doi:10.1038/mp.2013.65)

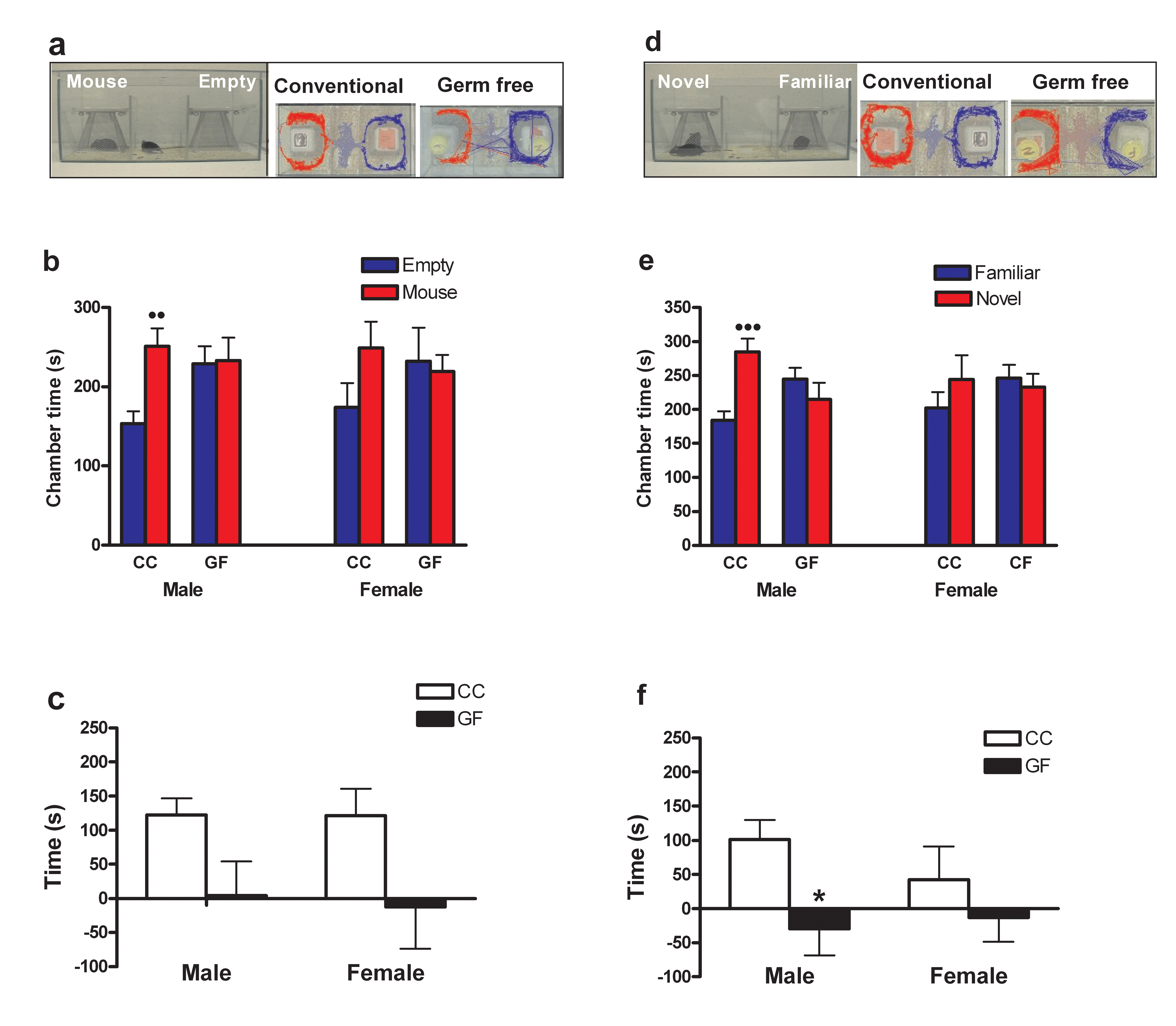

Supplement: Supplementary file 2 — Supplementary Figure 1 (JPG 5781 kb) [file 41380_2014_BFmp201365_MOESM346_ESM.jpg]

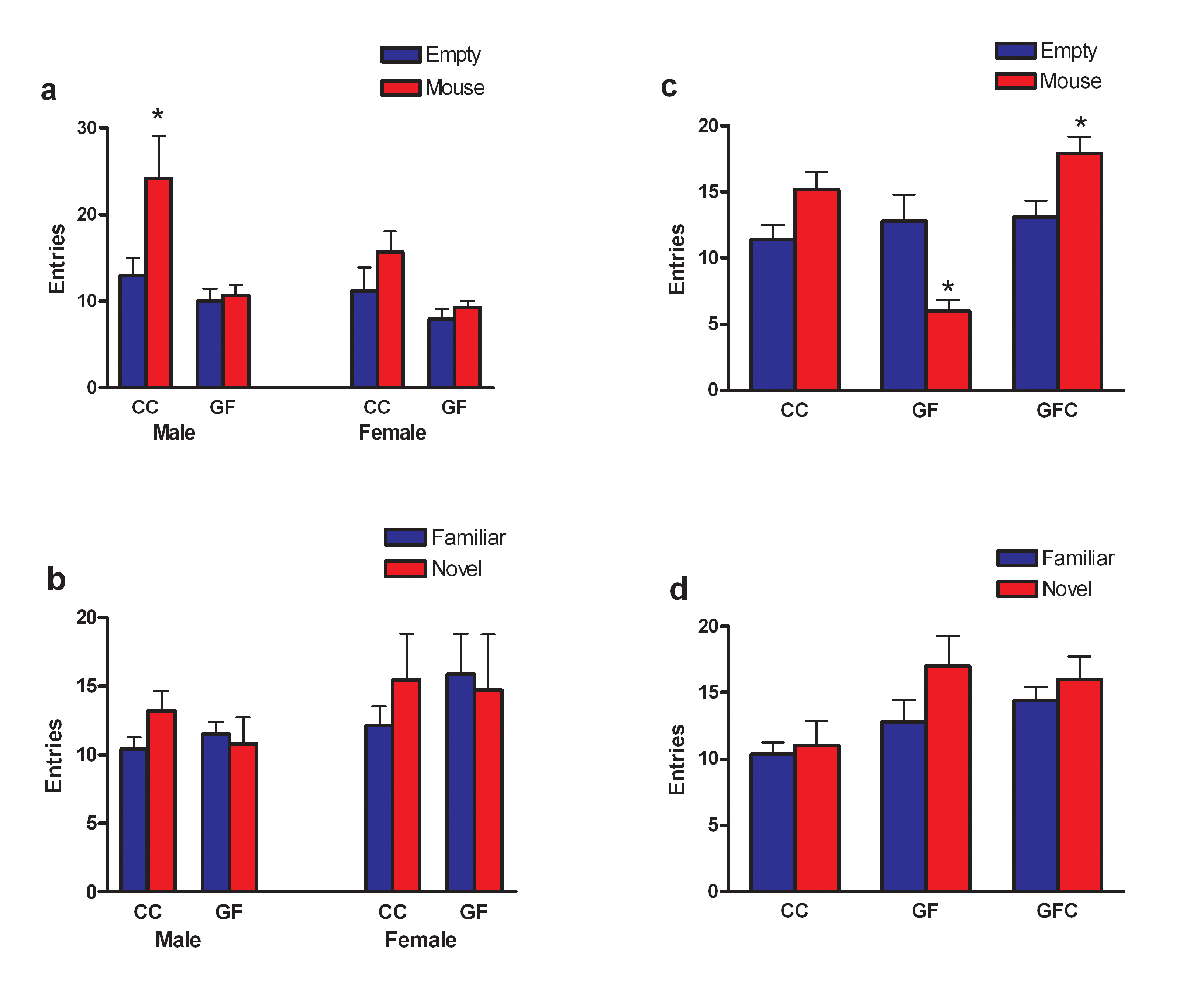

Supplement: Supplementary file 3 — Supplementary Figure 2 (JPG 1167 kb) [file 41380_2014_BFmp201365_MOESM347_ESM.jpg]

## Slide 1
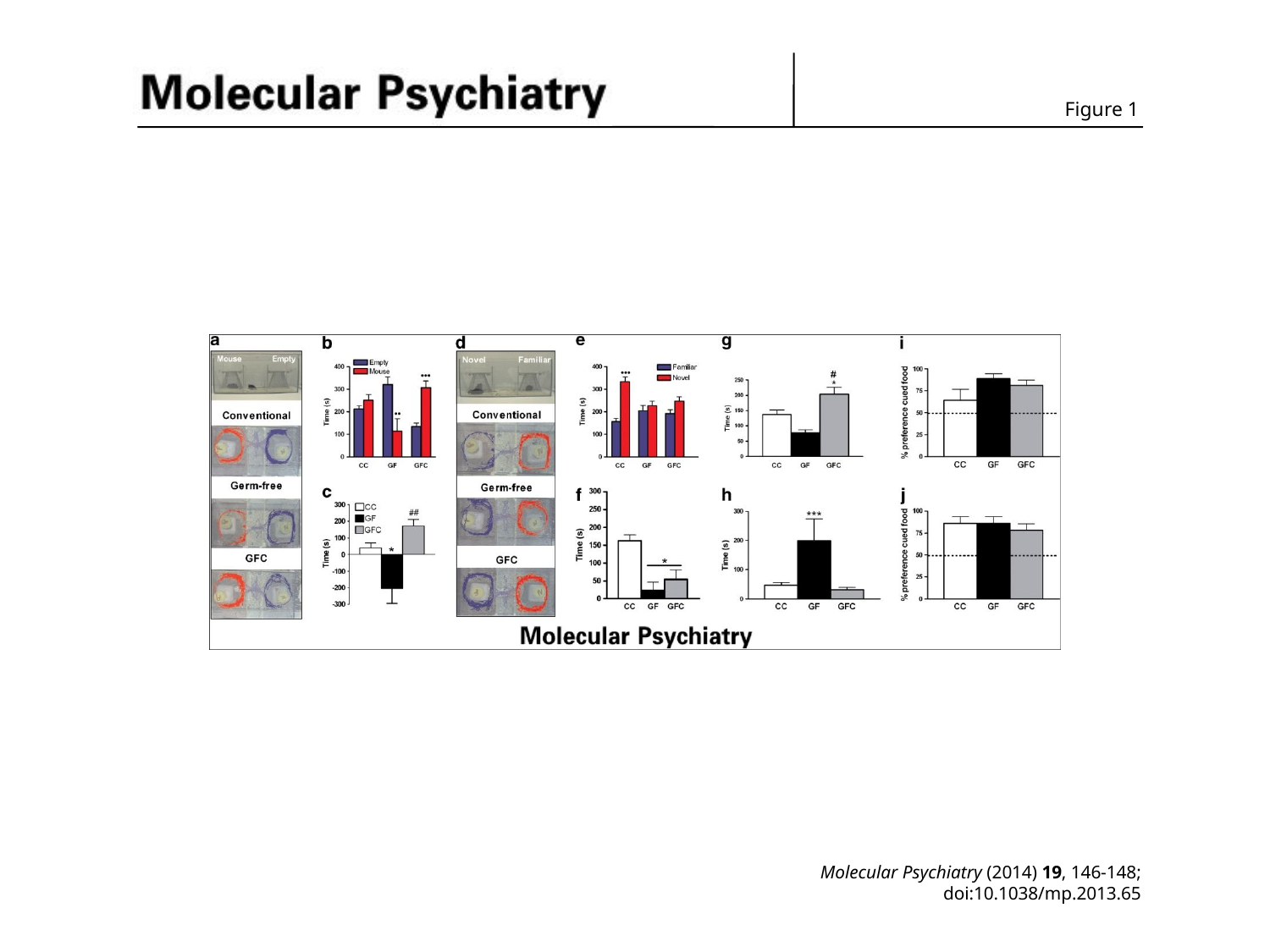

Figure 1
Molecular Psychiatry (2014) 19, 146-148;
doi:10.1038/mp.2013.65

Supplement: Supplementary file 4 — PowerPoint slide for Fig. 1 [file 41380_2014_BFmp201365_MOESM344_ESM.ppt]
